# How adults with treatment resistant depression experience their first esketamine nasal spray treatment? Preliminary results from a French qualitative study

**DOI:** 10.1192/j.eurpsy.2024.253

**Published:** 2024-08-27

**Authors:** E. Manolios, J. Mathé, J. Sibeoni, M. Rotharmel, B. Astruc, B. Falissard, L. Mekaoui, A. Laurin, E. Gaudre-Wattinne, J. Dupin, A. Revah-Levy

**Affiliations:** ^1^ECSTRRA team, INSERM; ^2^Service de Psychiatrie et Addictologie de l’adulte et du sujet âgé, Hôpital européen Georges-Pompidou , APHP; ^3^IPSEA, Paris; ^4^SUPADO, CH Argenteuil, Argenteuil; ^5^University Department of Psychiatry, Centre d’Excellence Thérapeutique, Centre Hospitalier du Rouvray, Rouen, France; ^6^Les Toises - Centre de psychiatrie et psychothérapie, Lausanne, Switzerland; ^7^CESP, INSERM U1018, Université Paris Saclay, Villejuif; ^8^CMME, GHU Paris Psychiatrie et neurosciences, Paris; ^9^CHU nantes, Nantes université, Nantes; ^10^Janssen Cilag, Issy les Moulineaux, France

## Abstract

**Introduction:**

Spravato® (esketamine nasal spray- ENS) is a new adjunctive drug for Treatment Resistant Depression (TRD), i.e. patients with major depressive disorder that failed to adequately respond despite the use of two different antidepressants. In France, a real world non-interventional post-commercialization cohort study is being conducted aiming to describe the conditions of use of the esketamine, and to observe the outcomes.

**Objectives:**

To in-depth explore the lived experience of first administered ENS treatment among adults with TRD, we are conducting an ancillary qualitative study.

**Methods:**

This qualitative study uses the IPSE approach (Sibeoni et al. *BMC Medical Research Methodology* 20.1(2020):1-21) and has been conducted in four French psychiatric departments. Design was based on the recruitment of patients through the Cohort study, all interviewed twice, the first time 3 to 5 weeks after the first administration of ENS, and the second time around 6 months after, whether treatment has been continued or not. Data analysis follows the IPSE analytic procedure and is conducted in two stages: three individual researchers carry out independent work and the group collectively pools data. These preliminary results are based on the sole analysis of the first interviews conducted from July 2022 to July 2023.

**Results:**

Eighteen participants with moderate to severe TRD, including 13 women, were interviewed and two axes of experience have been produced: (1) the overwhelming experiences of the treatment, perceived differently depending on patients, as a dissociative experience, both inside – described as a *trip-* and outside of them; (2) A discordant treatment experience with both solitude and relational support from medical team.

**Conclusions:**

These results highlight the need to better prepare the patients for the initiation of the treatment and to take into consideration the settings in which the treatment is administered, as well as the importance of the support received from the nursing staff.

**Disclosure of Interest:**

E. Manolios Grant / Research support from: have recieved financial support to conduct the study, J. Mathé Grant / Research support from: have recieved financial support to conduct the study, J. Sibeoni Grant / Research support from: have recieved financial support to conduct the study, M. Rotharmel Consultant of: Janssen, B. Astruc Consultant of: Janssen, B. Falissard Consultant of: Janssen, L. Mekaoui Consultant of: Janssen, A. Laurin Consultant of: Janssen, E. Gaudre-Wattinne Employee of: Janssen Cilag, J. Dupin Employee of: Janssen Cilag, A. Revah-Levy Grant / Research support from: have recieved financial support to conduct the study

